# Cross-Talk Between Gluten, Intestinal Microbiota and Intestinal Mucosa in Celiac Disease: Recent Advances and Basis of Autoimmunity

**DOI:** 10.3389/fmicb.2018.02597

**Published:** 2018-11-01

**Authors:** Atul Munish Chander, Hariom Yadav, Shalini Jain, Sanjay Kumar Bhadada, Devinder Kumar Dhawan

**Affiliations:** ^1^Department of Endocrinology, Postgraduate Institute of Medical Education and Research, Chandigarh, India; ^2^Department of Biophysics, Panjab University, Chandigarh, India; ^3^Center for Diabetes, Obesity and Metabolism, Wake Forest School of Medicine, Winston-Salem, NC, United States

**Keywords:** intestinal microbiome, oral microbiome, probiotics, glutenases, functional foods, host microbe interactions

## Abstract

Celiac disease (CD) is an autoimmune disorder of the small intestine, caused by gluten induced inflammation in some individuals susceptible to genetic and environmental influences. To date, pathophysiology of CD in relation to intestinal microbiota is not known well. This review relies on contribution of intestinal microbiome and oral microbiome in pathogenesis of CD based on their interactions with gluten, thereby highlighting the role of upper gastrointestinal microbiota. It has been hypothesized that CD might be triggered by additive effects of immunotoxic gluten peptides and intestinal dysbiosis (microbial imbalance) in the people with or without genetic susceptibilities, where antibiotics may be deriving dysbiotic agents. In contrast to the intestinal dysbiosis, genetic factors even seem secondary in disease outcome thus suggesting the importance of interaction between microbes and dietary factors in immune regulation at intestinal mucosa. Moreover, association of imbalanced counts of some commensal microbes in intestine of CD patients suggests the scope for probiotic therapies. *Lactobacilli* and specific intestinal and oral bacteria are potent source of gluten degrading enzymes (glutenases) that may contribute to commercialization of a novel glutenase therapy. In this review, we shall discuss advantages and disadvantages of food based therapies along with probiotic therapies where probiotic therapies are expected to emerge as the safest biotherapies among other in-process therapies. In addition, this review emphasizes on differential targets of probiotics that make them suitable to manage CD as along with glutenase activity, they also exhibit immunomodulatory and intestinal microbiome modulatory properties.

## Introduction

Celiac disease is a common autoimmune disorder of the small intestine and is manifested by intestinal inflammation, destruction of intestinal villi (villous atrophy), elongated crypts (crypt hyperplasia) (Schuppan et al., 2009) and altered intestinal barrier (Schulzke et al., 1998). CD was first reported to have prevalence 1: 184 in an Italian scholar population (Catassi et al., 1996). Prevalence of CD varies from 0.006 to 5.6% across different populations of world. As per recent reports, prevalence of CD in United States is 0.79% (Mardini et al., 2015). In European countries, its prevalence is 4% in Spain (Cilleruelo et al., 2015), 2.4% in Finland (Mustalahti et al., 2010), 0.9% in Germany (Laass et al., 2015), 0.6% in Northern Sweden and Hungary (Enroth et al., 2013; Burger et al., 2014), lesser in Scotland 0.01% (White et al., 2013) and least in Netherlands 0.006% (Burger et al., 2014). In Asian countries, prevalence of CD is 0.3% in Iran, 0.5% in Turkey, 0.7% in Israel (Shamir et al., 2002; Singh et al., 2015) and 0.73% across different regions of India (Ramakrishna et al., 2016). Saharawi population of Africa accounts 5.6% as the highest prevalence worldwide (Teresi et al., 2010). The presence of variant forms of human leukocyte antigen (HLA) genes are reported to be associated with the intestinal inflammation in CD but all types of genetic variations could define 48% of the disease risk thereby suggesting some other factors associated with its pathogenesis (Gutierrez-Achury et al., 2015). The extent of gluten intake is strongly associated with prevalence of CD rather than HLA genetics (Ramakrishna et al., 2016), but as gluten itself does not explain the reason for increased disease incidence and disease progression, thus environmental factors other than gluten need to be addressed (Pozo-Rubio et al., 2012; Lerner et al., 2015). In context with current scenario, intestinal dysbiosis is well reported in CD besides certain environmental factors including microbiota composition especially in infants are also associated with CD (Pozo-Rubio et al., 2013). Further, intestinal microbial ecology of commensals along with symbiotic and pathogenic microorganisms play an important role in pathogenesis of many gastrointestinal diseases. Intestinal bacteria interfere with the mammalian immune system and regulate differentiation of pro-inflammatory and anti-inflammatory T-cells through many pathways, among which TLR pathway (Round and Mazmanian, 2009; Cerf-Bensussan and Gaboriau-Routhiau, 2010) is a key factor for integrity and functionality of the tight junction barrier of the intestinal epithelial layer (Honda and Littman, 2012).

Even for several decades, CD has been known as a complex autoimmune disease. Despite knowledge about antigenic trigger, there is no successful therapy available to alter the option of Gluten Free Diet (GFD). Strict lifelong adherence to GFD always remains a challenge for patients. Further, unintentional ingestion of gluten contaminated food facilitates reoccurrence of gluten induced inflammation because intestinal microbial balance is not restored even after being on GFD (Nadal et al., 2007; Collado et al., 2008, 2009). This is suggestive of persistent intestinal environment which is disease susceptible and does not allow recovery of intestinal mucosa. Probiotics are beneficial in a number of gastrointestinal disorders (Cheng et al., 2013; de Meij et al., 2013; Sanchez et al., 2013). This review lays emphasis on the role of oral and intestinal microbiota in pathogenesis of CD, and also elaborates on the beneficial effects of some intestinal microbes in immunomodulations as well as in detoxification of immunogenic gluten peptides. Though, genetic factors are important in pathogenesis of CD but environmental factors serve as crucial triggers for disease onset and progression.

## Genetic Associations of CD

Involvement of HLA genes in the pathogenesis of CD is quite imminent and thus explains the risk associated with the disease. Recently, in a meta-analysis, De Silvestri et al. (2018) has reported that HLA-DQ genetics can even be used as a genetic screening procedure for CD children (De Silvestri et al., 2018). Studies across the world explain that about 40% of the risk associated with this disease depends on HLA class II region contributing to CD. Most CD subjects are HLA-DQ2 (DQA1^∗^05/DQB1^∗^02) positive (90%), while half of the remaining 10% are HLA-DQ8 (DQA^∗^0301/DQB1^∗^0302) positive (Sollid et al., 1989). Genetic risk due to HLA genes in CD is due to the high binding affinity of gluten peptides with DQ2/DQ8 alleles of HLA molecules.

Disease risk varies depending upon the ethnicity thereby signifying the importance of environmental factors. Involvement of risk genes in the disease pathogenesis varies with ethnicity as reported (Alshiekh et al., 2017). This study has shown that in a subpopulation analysis, DRB3^∗^01:01:02-DQA1^∗^ 05:01-DQB1^∗^02:01 remained the most significant in patients with Scandinavian ethnicity whereas DRB1^∗^07:01:01-DRB4^∗^01:03:01-DQA1^∗^02:01-DQB1^∗^02:02:01 presented the highest risk of CD among non-Scandinavians. Thus, it was interpreted that different DRB1^∗^03:01-DQB1^∗^02:01 haplotypes confer different risks for CD. The associated risk of CD for DR3-DRB3^∗^01:01:02-DQA1^∗^05:01-DQB1^∗^02:01 is predominant among patients of Scandinavian ethnicity.

Studies in India have shown the association of HLA genes with Type 1 diabetes as well as CD (Serjeantson et al., 1987; Agrawal et al., 2000; Kaur et al., 2002; Kumar et al., 2007, 2012; Mehra et al., 2007; Raha et al., 2013). Amarapurkar et al. (2015), reported that CD in Indian patients is predominantly associated with HLA DQ 2 and DQ 8 genotypes and has high positive predictive value for diagnosis when combined with serology in symptomatic patients (Amarapurkar et al., 2015). While, validating the European risk loci for CD in 497 cases and 736 controls of north Indian origin, Senapati et al. (2015), have reported that the north Indian population has a higher degree of consanguinity than Europeans and therefore explored the role of recessively acting variants, which replicated the HLA locus and suggested a role of additional four loci (Senapati et al., 2015).

In a study performed by Singla et al. (2016), of 202 first-degree relatives of the 64 index cases with CD, 17.3% (35/202) were seropositive for IgA tTG while biopsy proven CD was diagnosed in 10.2% (8/78) of children and 8.1% (10/124) of adults. HLA DQ2/DQ8 was positive in 96.7% of the index cases and all first-degree relatives with confirmed CD (Singla et al., 2016). Another study conducted on a cohort of first degree relatives by Mishra et al. (2016), has shown that prevalence of CD in first-degree relatives of CD patients was 10.9% and 87% had HLA DQ2 or DQ8 haplotype, thus concluding that all first-degree relatives of CD patients should be screened for CD even if associated with asymptomatic or with atypical manifestations. Srivastava et al. (2010), in their first Asian study on a limited number of families of children with CD have reported that 4.4% of the first-degree relatives had CD whereas only 15% of the first-degree relatives were negative for HLA DQ2/DQ8 (Srivastava et al., 2010) and thus suggested the importance of other genetic or environmental factors in disease pathogenesis.

## Microbes in Pathogenesis of CD

**FIGURE 1:**
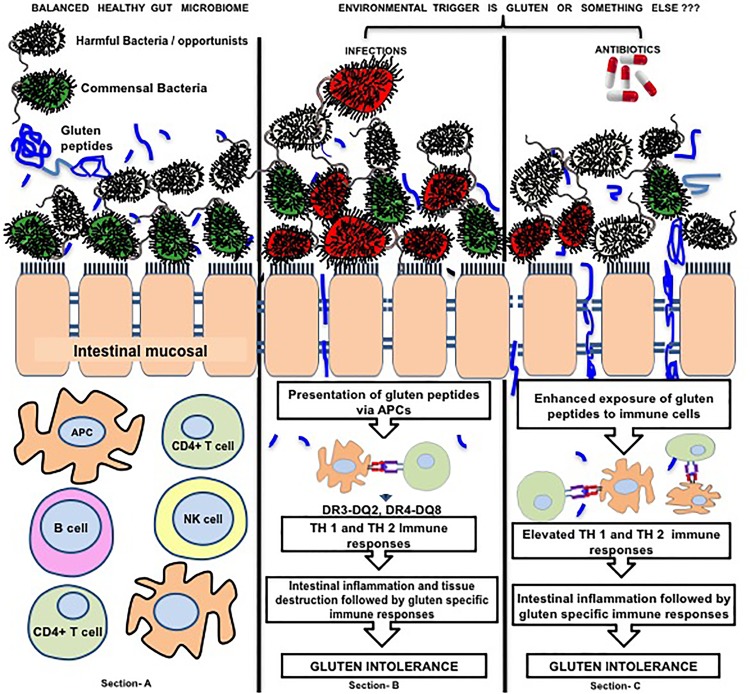
Figure represents the interaction of gluten, microbes and intestinal immune regulation. Section (A) of the image represents healthy microbial ecology where there is a balance between beneficial bacteria (green) and harmful bacteria (red yellow) and beneficial microbes preventing the adhesion of harmful ones to intestinal mucosa. Section (B) represents exposure to environmental infectious agents (red) that compete with the beneficial microbes to adhere the intestinal mucosa and after adherence they disturb intestinal barrier function (tight junctions) by activating different inflammatory pathways at intestinal mucosal surface. Disturbed intestinal barrier leads to exposure of intestinal immune cells to the dietary antigens (i.e., gluten). On the other hand, infections possess elastase activity to the peptides that are more potent to translocate through intestinal barrier. These gluten peptides (blue colored) are presented by APCs to the T lymphocytes leading to cascades of immune processes leading to gluten specific immune responses and tissue remodeling to develop gluten intolerance. Section (C) highlights that the catalyst role of antibiotics that are given to patients to treat infection. Antibiotics eradicate infections along with beneficial intestinal microbes thereby leading to dysbiosis. In this condition, the opportunistic harmful microbes may prove to be dangerous because beneficial microbes can no longer protect intestinal mucosa leading to adherence of the former to the intestinal epithelial cells and creating disease susceptible microenvironment (25). Color of beneficial microbes, Green; Gluten peptides, blue colored; Color of Harmful/ Opportunist microbes,Gray colored; Color of Infections, Red colored. The arrows marking downward represent decrease in event whereas arrows marked upward represent increase in an event.

Microbiome dysbiosis in CD is reported by several studies (Sellitto et al., 2012). In a pursuit to explore the role of HLA genes in pathogenesis of CD, a recent report of 22 individuals genetically at risk of CD concluded that HLA-DQ2 genotype selects early intestinal microbiota composition when compared to individuals at lower genetic risk/ carriers. The individuals at high risk showed different intestinal microbiota i.e., high-risk infants had significantly less *Bifidobacteria* and unclassified *Bifidobacteriaceae* proportions and more *Corynebacteria, Gemella, Clostridium sensustricto*, unclassified *Clostridiaceae*, unclassified *Enterobacteriaceae* and *Raoultella* proportions (Olivares et al., 2015). On the other hand, a recent Indian study on 23,331 adults supported the importance of other factors rather than genetics because HLA genes were not associated with prevalence of CD (Ramakrishna et al., 2016) but the extent of gluten was. An association of microbiota with CD was first established in GFD T-CD and U-CD subjects (Nadal et al., 2007; Collado et al., 2008, 2009) and thus a concept of dysbiosis was put forward. An Italian study reported that intestinal infections were strongly associated with the onset of disease and were further strongly associated with antibiotics use (Canova et al., 2014). Moreover, early age infections and infants’ antibiotic intake is also reported as the cause of dysbiosis and alterations in lymphocyte subpopulations (Pozo-Rubio et al., 2013) that can be correlated to disease activity i.e., increased cellularity (increase in number of intraepithelial lymphocytes) and atrophy of small intestinal mucosa, a characteristic feature of CD (Shmidt et al., 2014). Such antibiotic induced dysbiosis was characterized by decreased counts of *Bifidobacterium longum* and increased counts of *Bacteroides fragilis* (Pozo-Rubio et al., 2013). Moreover, dose-response relationship of antibiotics is significantly associated with onset of CD and risk of CD is further increased by cephalosporin intake (Canova et al., 2014). In contrast, several previous studies reported that dysbiosis in CD is characterized by decrease in *Bifidobacteria* counts (Sanz et al., 2007; Di Cagno et al., 2009; Golfetto et al., 2014; Giron Fernandez-Crehuet et al., 2015), pointing on antibiotics and infections as the key players of dysbiosis that may be a reason of disease susceptibility. In such a situation, a likely question arises that “Environmental trigger is only gluten or there is something else too i.e., microbiome dysbiosis, infections and antibiotics, or antibiotic induced dysbiosis.” The underlying hypothesis has been represented in the Figure 1. Intestinal microbial overgrowth is characteristic of CD and specific pathobionts namely *Klebsiella oxytoca, Staphylococcus epidermidis*, and *S. pasteuri* were reported to outnumber commensals and they have potential to exclude commensals from intestine (Sanchez et al., 2013). CD is a T-cell mediated disease in which gliadin-derived peptides cause inflammatory actions at intestinal epithelium thereby affecting lamina propria and T lymphocytes. Activation of T lymphocytes and other immune cells further leads to the release of proinflammatory cytokines IFN-*γ* and IL-15 that are responsible for the activation of the cytotoxicity in intraepithelial lymphocytes (Gianfrani et al., 2005; Meresse et al., 2009). Studies thus suggest the imperative interaction of intestinal bacteria with immune system to direct the differentiation of both pro-inflammatory and anti-inflammatory T cell populations (Round and Mazmanian, 2009). The role of Regulatory T cells (Treg) has become clearer with the efforts of Serena et al. (2017) that help us to understand the pathogenic role of gut microbiota and their metabolites in CD through epigenetic processes. Treg cells are subset of CD4 T cells responsible for maintaining immune response to foreign antigens (Lehtimäki and Lahesmaa, 2013). Treg cells mediate suppression of responder cells by different mechanisms (Pellerin et al., 2014; Shevach, 2018). Previous studies have reported an increase in the number of Treg cells in CD patients and suggested that functional impairments in their suppressive function may be related to the onset of the disease (Cook et al., 2017). The exact mechanisms that cause the altered regulatory activity of Treg cells in CD is not known. FoxP3 is a transcription factor fundamental for the suppressive function and differentiation of Treg cells. In a recent report, a higher expression of an alternatively spliced isoform of FOXP3 (FOXP3 Δ2) was observed in the intestine of active CD patients as compared to non-celiac controls while no differences were seen in the expression of FOXP3 full length (FL). It suggests that altered intestinal ratio between FoxP3 isoforms may be an important indices in the pathogenesis of CD. Furthermore, higher expression of isoform FOXP3 Δ2 is reported to be associated with inflammation. *Ex-vivo* cultures of intestinal biopsies treated with butyrate trigger a balance between FoxP3 isoforms in HC subjects, while the same does not occur in CD patients. That shows the role of microbial metabolites in modulation of splicing, thus triggering to an inflammatory state with some role in pathogenesis of CD (Serena et al., 2017).

The increased expression of altered spliced from of FOXP3 was correlated to butyrate dependent altered intestinal micro-environment in CD patients. To the best of knowledge in the current literature, there is not a single report related to small intestinal butyrate levels of CD vs. controls. Serena et al. (2017) have co-related their small intestinal biopsy based concept with fecal metabolome based reported studies that looked a little controversial toward development of their hypothesis. Microbial derived metabolite in feces is not a reflection of those from small intestine because microbial communities and microbial derived metabolites differ in different parts of gut (Gorham et al., 2017). In this line of research, levels of butyrate in small intestine is pending to be evaluated in comparison to controls subjects. On the other hand, beneficial and detrimental effects of butyrate on the mucosal immune system are basically concentration dependent (Jiminez et al., 2017) and the same was not ruled out for *in-vitro* experiments by Serena et al. (2017). Thus, at this time point, the hypothesis of detrimental effects of butyrate in CD which are mediated through splicing, may require further work.

### Mucosal Immunology of CD

Intestinal barrier function is associated with many autoimmune diseases and CD. Zonulin modulates the intercellular tight junctions and reversibly regulates permeability of intestine to luminal antigens and other components (Wang et al., 2000). Increased gene expression of zonulin is reported in CD and T1D, relating to tight junction barrier dysfunction (Fasano et al., 2000; Sapone et al., 2006). Microbes and gliadin are two important factors that upregulate the expression of zonulin leading to zonulin release from the cells (Sturgeon and Fasano, 2016). Administration of gliadin to the intestinal cells causes zonulin release followed by an increase in permeability. Binding of some gliadin peptides to CXCR3 receptor of enterocytes causes MyD88-dependent zonulin release. Gliadin and exposure to bacteria work in a similar manner to cause release of pro-inflammatory cytokines and zonulin from macrophages (Thomas et al., 2006).

Due to various etiologies, infections are important in pathogenesis of some gastrointestinal diseases. Intestinal microbes interact with immune cells and activate the inflammatory cascades through TLR pathway (Round and Mazmanian, 2009; Cerf-Bensussan and Gaboriau-Routhiau, 2010) that regulates the intestinal barrier function (Honda and Littman, 2012). The interaction of intestinal epithelial cells takes place with microbes residing in the gut lumen via pattern recognition receptors such as TLRs and Nod-like receptors that induce antibacterial molecules and chemokines to promote adaptive immune responses in the intestine (Cerf-Bensussan and Gaboriau-Routhiau, 2010). Moreover, TLR2 pathways have been reported to be involved in intestinal barrier function as mice lacking TLR2 expression are more susceptible to microbial-induced colitis (Honda and Littman, 2012). In addition, duodenal TLR2 expression is lower in CD subjects and higher for TLR9 as compared to HCs (Kalliomaki et al., 2012) thereby suggesting the involvement of microbes in inducing impairment of intestinal barrier function and immune dysregulation through TLR dysfunction. Furthermore, microorganisms stimulate IFN-γ, TNFα production and activate T-cell macrophage-associated activity that may trigger a TH1-type cytokine profile (Li et al., 2008). Such pro-inflammatory response can contribute to an increase in the epithelial permeability that favors the access of higher antigen loads to the submucosa (Li et al., 2008; Menard et al., 2010; Johnston and Corr, 2016). Thus, intestinal bacteria can also regulate the ability of monocytes recruited to the mucosa to respond to gliadins and IFN-γ in CD subjects, thereby influencing the course of the disease (De Palma et al., 2010). The process of autoimmunity might have resulted in epithelial stress triggered by infections, gluten peptides and inflammation. Some experimental studies further affirm the interactions between gluten, microbiota and mucosal immune dysregulation.

The composition of the intestinal microbiota affects the permeability of the intestinal mucosa and could be involved in the early stages of CD development (Cinova et al., 2011). Some CD infections like *Pseudomonas aeruginosa* directly trigger the process of inflammation as reported by Caminero et al. (2016). *P. aeruginosa* possess elastase properties to cleave gluten peptides in such manner making them highly immunotoxic. These peptides were more potent to be translocated through the intestinal barrier and to cause activation of T-cells from CD patients (Caminero et al., 2016). In context to pathogenic role of some intestinal microbes in CD, simultaneous exposure of Caco-2 cells to *Bacteroides fragilis* and gliadin cause an increase in intestinal permeability as well as increased production of TNFα and IL-1β18 (Sanchez et al., 2012). After *ex-vivo* treatment of duodenal biopsy with gluten digest and CD associated bacteria, IL-17A responses were induced (Sjoberg et al., 2013) that are associated with pro-inflammatory cytokines in autoimmune and inflammatory diseases (Costa et al., 2010). Sequencing technologies provide the basis of host microbe interactions. Whole genome sequencing of microbes has enabled to predict pathogenic behavior of microbes (Chander et al., 2016a,b; D’Argenio et al., 2016) whereas D’Argenio et al. (2016) confirmed the pathogenic role of CD gut microbes by combination of *in-vitro* approaches. As they revealed that *Neisseria flavescens* species were most abundant in the symptomatic patients of CD. Genetic composition of virulence determinant genes was different in the strains of HCs compared to the diseased ones, as showed by whole genome sequence of the microbes. In addition, these strains isolated from patients, activated inflammatory responses in DCs and in *ex-vivo* culture of duodenal biopsies (D’Argenio et al., 2016).

As the process of immune dysregulation in CD is looking to be derived from intestinal dysbiosis and infections while antibiotics act as trigger giving rise to a disease prone dysbiosis. Antibiotics not only irradiate enteric infections but are also known to exert harmful impact on beneficial gut microbes thereby causing intestinal dysbiosis. The dysbiotic microbial texture generally produces a disease prone environment. The following sections describe the types of dysbiosis caused in CD and its impact on commensal organisms.

### Intestinal Dysbiosis in CD

In some studies, dysbiosis in CD subjects was represented by outnumbers of *Bacteroides* spp. and lower numbers of *Bifidobacterium spp*. and *Bifidobacterium longum*, that remained unchanged after GFD (Nadal et al., 2007; Collado et al., 2008, 2009). Other studies reported increased prevalence of *Bifidobacterium dentium* and *Bacteroides vulgatus* while decreased prevalence of *Bifidobacterium catenulatum* and *Bacteroides species* (Collado et al., 2008; Sanchez et al., 2010, 2011). During analysis of fecal microbiota in active/ symptomatic (patients presenting gastrointestinal symptoms) CD children, the levels of *Bacteroides, Clostridium, Staphylococcus, C. histolyticum*, Prevotella, *Eubacterium rectal, C. coccoides*, Atopobium, and sulfate reducing bacterial groups were reported higher (Collado et al., 2007). Greater prevalence of infectious microbes and gram-negative bacteria in the duodenum of celiac children was found responsible for symptomatic presentation of the disease (Nadal et al., 2007). Analyzing duodenal microbiota, T-CD patients with persistent symptoms occupy different textures of intestinal microbes in comparison with those without symptoms. Patients with persistent symptoms comprised of higher relative abundance of *Proteobacteria* and a lower abundance of *Bacteroidetes* and *Firmicutes.* These patients presented persistent symptoms even after following strict GFD. Comparative microbial richness in these patients was observed lesser in comparison to control subjects (Wacklin et al., 2014).

As per a recent report, when infants at high genetic risk were compared to those with lower risk, lower proportions of duodenal *Actinobacteria* and *Bifidobacteria* were observed in the former whereas higher proportions of *Firmicutes* and *Proteobacteria* were found in the later (Olivares et al., 2015). Some studies showed direct relationship of commensal bacterial groups indicating the potential therapeutic implications as described in the next sections.

### Associations of *Lactobacilli* and *Bifidobacteria* With CD

Based on the recent advancements in the molecular studies, we can clearly find a strong association of some commensal bacterial groups with CD that may conjecture about the modulation of pathogenesis of disease by them.

In some new findings, significant lower counts of *Lactobacilli* were found in feces of celiac children on GFD as compared to healthy children, whereas *Enterobacteria* were found increased in celiac children (Lorenzo Pisarello et al., 2015). Compared to the healthy subjects, fecal counts of *Bifidobacteria* were significantly higher in T-CD patients of Brazil (Golfetto et al., 2014). While studying the duodenal microbiota, counts of *Lactobacillus* were found significantly reduced as compared to the healthy individuals. CD patients were possessing *Streptococcus, Bacteroides* and *E.coli* species whereas lower counts of *Streptococcus* and *Bacteroides* were observed and numbers of *Bifidobacteria, Lactobacillus* and *Acinetobacter* were found higher in controls (Giron Fernandez-Crehuet et al., 2015).

The ratio of *Lactobacilli* and *Bifidobacteria* to *Bacteroides* and *Enterobacteria* was lesser in T-CD subjects as compared to HCs and this difference was more when compared with U-CD subjects (Di Cagno et al., 2009, 2011).

In contrast, the GFD fed CD subjects have also shown a reduction in the diversity of *Lactobacillus spp.* and *Bifidobacterium spp.* while presence of *Bifidobacterium bifidum* was higher in normal diet fed CD subjects than healthy adult (Nistal et al., 2012). *Bacteroides* and *Escherichia coli* were significantly abundant in CD patients that presented marked gastrointestinal symptoms than in controls and asymptomatic patients (Nadal et al., 2007). The ratio of beneficial microbes (*Lactobacillus* and *Bifidobacterium)* to harmful ones (*Bacteroides* and *E. coli)* was reported significantly lower in symptomatic and asymptomatic diseased subjects compared with controls (Nadal et al., 2007). *Leuconostoc mesenteroides, Lactobacillus curvatus*, and *Leuconostoc carnosum* species were reported as characteristic of CD subjects, while *Lactobacillus casei* group was characteristic of HCs while species diversity for *Bifidobacterium* was significantly higher in healthy children than that of CD (Sanz et al., 2007). It would be appropriate to conclude that decreased numbers and types of *Lactobacilli* and *Bifidobacteria* strains are associated with CD and may have influence in the outcome of the disease. The results derive their implications toward therapeutic use because *Lactobacilli* are recently well proved for their glutenase activity and anti-inflammatory actions (Di Cagno et al., 2004; De Angelis et al., 2006; Duar et al., 2014).

## Interaction of Microbiota and Gluten: a Basis of Enzyme Therapy and Glutenase Therapy

Complete digestion of gluten proteins (gliadins and glutenins) is difficult by human proteolytic enzymes (Wieser, 2007). These proteins have been reported to possess differential immune targets that make them immunotoxic in nature (Caputo et al., 2012; Capozzi et al., 2013; Iacomino et al., 2013).

During proteolytic digestion in intestine, proline and glutamine rich gluten polypeptides are produced that are immunogenic and have the potential to stimulate T cells (Sollid and Khosla, 2005). These peptides are resistant to further hydrolysis due to enrichment of proline residues in the amino acid sequences (van de Wal et al., 1999; Arentz-Hansen et al., 2000; Shan et al., 2002; Molberg et al., 2003; Dewar et al., 2006; Siegel et al., 2006; De Vincenzi et al., 2010; Mamone et al., 2013). As most of the peptides are immunogenic in nature, some of them (p10-mer, QQPQDAVQPF) possess protective effects whereas others prevent the gliadin-dependent dendritic cell maturation (Giordani et al., 2014).

Some new studies further highlighted the value of gut microbes in determination of gluten immunogenicity. *P. aeruginosa* isolated from CD patients cleaved 33 mer peptide in such a manner that it activated gluten-specific T-cells in CD patients. On the other hand, *Lactobacillus* spp. isolated from the non-CD controls had potential to reduce the immunogenicity of the peptides produced by *P. aeruginosa* (Caminero et al., 2016). Looking at the necessity of degrading such peptides, an era of glutenases seems an evergreen field of research for years.

### Trends in Glutenases

Some studies were targeted to explore the proteolytic enzymes from sources other than bacteria that could degrade the immunogenic gliadin. An enzyme from barley, EP-B2 (glutamine-specific endoprotease) degrades complex gluten bread proteins whereas PEP from *Sphingomonas capsulata* detoxifies the residual oligopeptide products of EP-B2 prolyl endopeptidase. AN-PEP is also known to enhance the gluten digestion in such an excellent manner that only traces of gluten were detected in small intestine (Gass et al., 2007; Mitea et al., 2008). AN-PEP degrades T cell stimulatory peptides and gluten (Stepniak et al., 2006). For still better digestion of gluten, proline and glutamine specific endopeptidases from barley, fungi and bacteria were taken into account collectively by different scientific communities (Shan et al., 2004; Siegel et al., 2006; Stepniak et al., 2006; Cerf-Bensussan et al., 2007; Bethune and Khosla, 2012).

The use of PEPs proved extensively effective in *in-vivo* (animal models) and *in-vitro* studies (Hausch et al., 2002; Shan et al., 2002; Marti et al., 2005). Encouraged from these preliminary studies, present microbiota research is more emphasized on discovery of gluten degrading bacteria that might help in enzyme therapy or directly as live culture against CD.

### Role of Oral to Intestinal Microbiota in Gluten Degradation

The oral cavity occupies protease producer microorganisms capable to hydrolyze proline and glutamine rich peptides (Helmerhorst et al., 2010; Zamakhchari et al., 2011). Salivary microorganisms exhibit glutamine endoprotease activity to degrade most of the gliadins (Helmerhorst et al., 2010).

*Rothia aeria* HOT-188, *Rotia mucilaginosa* HOT-681, *Streptococcus mitis* HOT-677, *Streptococcus* sp. HOT-071, *Actinomyces odontolyticus* HOT-701, *Neisseria mucosa* HOT-682 and *Capnocytophaga sputigena* HOT-775 were the other gluten degrading bacteria identified by Fernandez-Feo et al. (2013). *R. mucilaginosa* and *R. aeria* are highly active toward gluten and are able to cleave 33-mer and 26-mer immunogenic peptides. Interestingly, the enzyme produced by *R. aeria* is active over a wide pH range (pH 3–10) of intestinal pH (Zamakhchari et al., 2011).

In addition, salivary microbiota and metabolome are reported to be associated with CD (Francavilla et al., 2014). Despite of significance of oral microbiota in gluten degradation, Caminero et al. (2014), gained attention by certifying the significance of intestinal/ duodenal microbiota in metabolism of proteins that is also a valuable work. *Lactobacillus helveticus* is known to efficiently cleave the long immunogenic peptides (Chen et al., 2003). Caminero et al. (2014) reported 144 strains of 35 bacterial species exhibiting the property of gluten metabolism. Most of these strains were from phyla Firmicutes and Actinobacteria. Exploring the role of duodenal microbes in gluten degradation, their group discovered 31 strains with extracellular proteolytic activity against gluten and 27 other strains had peptidolytic activity against the 33-mer peptide (Herran et al., 2017).

A mixture of *Lactobacilli* and fungal proteases were studied to demolish the immunogenicity of wheat by long-time fermentation and 33-mer peptide was efficiently hydrolyzed by *Lactobacilli* (Rizzello et al., 2007).

On the footsteps of traditional trends in glutenases, modern research is promoting to commercialize the glutenases into action. Savvateeva et al. (2015), proved that recombinant wheat cysteine protease triticain-α exhibited glutenase and collagenase activities that is stable at intestinal pH and was potent to hydrolyze immunotoxic gluten peptides. In a phase 2 trial, glutenase ALV003 successfully attenuated mucosal injury in CD patients consuming upto 2 g gluten daily along with GFD (Lahdeaho et al., 2014). Approaches to screen gluten degrading bacteria are still in fast progression. *Lactobacillus ruminis, Lactobacillus johnsonii, Lactobacillus amylovorus, Lactobacillus salivarius* bacteria comprises of high peptide-degrading properties. All the strains possessed different degradation rates and cleavage patterns capable of reducing immunotoxic gluten peptides but were not efficient for complete removal of peptides (Duar et al., 2015).

## Gut Flora Modulation: Impact on CD

Now CD is known for overgrowth of pathogenic microbes that dominates to symbionts thereby excluding the later from intestinal ecosystem (Sanchez et al., 2013). Administration with some probiotics either alters the intestinal microbiome composition or brings about some health beneficial immunomodulations. As described here, the gut flora modulation proved to be beneficial when trials were conducted in different *in-vitro* and *in-vivo* models and somehow in human trials also.

### Probiotics

Probiotics are live microorganisms that provide health beneficial impact on host when administered in adequate amounts (Guarner et al., 2005). Reports showing probiotic induced beneficial effects in animal models of CD certify that probiotics have a positive influence on disease pathology through different mechanisms. Down-regulation of pro-inflammatory biomarkers, expression of NF-kB, TNF-α, and IL-1β was modulated in cell culture experiments after administration with *Bifidobacteria* (Laparra and Sanz, 2010; Table 1).

**Table 1:** Differential mode of action of probiotics in gluten induced immunotoxicity.

Probiotic used	Model used	Mode of sensitization	Key findings	References
*Bifidobacterium longum* CECT 7347	Female, weaning Wistar rats	IFN-*γ* and fed gliadin	*Bifidobacterium longum* attenuates the production of inflammatory cytokines and the CD4 + T-cell mediated immune response.	Laparra et al., 2012
*Lactobacillus casei*	Transgenic mice expressing the human DQ8 heterodimer	Chymotryptic digest of gliadin along with cholera toxin	Enhanced the gliadin specific response mediated by CD4 + T cells.	D’Arienzo et al., 2008
*Saccharomyces boulardii* KK1 strain	BALB/c mice	Gluten-containing commercial food pellets	Improved enteropathy development in association with decrease of epithelial cell CD71 expression and local cytokine production.	Papista et al., 2012
*Bifidobacterium longum* CECT 7347	Female weanling Wistar rats	Gliadin	Ameliorate the inflammation caused by gliadin.	Olivares et al., 2012
*Lactobacillus casei*	Transgenic mice expressing the HLA-DQ8 molecule in the absence of endogenous mouse class II genes, non transgenic for human CD4.	Wheat gliadin	*L. casei* can be effective in rescuing the normal mucosal Architecture.	D’Arienzo et al., 2011


### Probiotic Based Approaches and Tolerance Induction

A strategy used to induce suppression of immune responses to an antigen to develop tolerance for the same antigen is known as tolerance induction. Different approaches for tolerance induction were used. Recombinant alpha-gliadin protein was used for the potential immunomodulation of this disease. Intranasal administration of recombinant alpha-gliadin in DQ8 transgenic mice induced downregulation of immune responses against gliadin (Senger et al., 2003). Another study was emphasized on oral tolerance that is the induction of antigen-specific suppression of immune responses to an antigen by its prior oral feeding. *Lactobacillus lactis* was genetically engineered for secretion of a DQ8 specific gliadin epitope. Oral administration of this bacteria suppressed DQ8 restricted T-cell responses in NOD AB° DQ8 transgenic mice (Huibregtse et al., 2009). This might be a promising therapeutic approach for treatment of CD and may prove to be helpful to prevent CD in DQ8 associated genetically susceptible individuals.

*Saccharomyces boulardii* KK1 strain had exhibited capability to hydrolyse the 28-kDa alpha-gliadin fraction, and when fed to mice, attenuated enteropathy development as well as caused decreased expression of epithelial cell CD71 + cells and cytokines (Papista et al., 2012). However, administration of the *Bifidobacterium longum* CECT 7347 alone to rats fed gliadins ameliorated the inflammation caused by gliadin feeding (Olivares et al., 2012). Recently, Alvarez-Sieiro et al. (2014), designed two food-grade *Lactobacillus casei* strains by genetic engineering that could deliver PEP. Out of these two, one was capable to secrete PEP into the extracellular medium whereas the other was able to maintain PEP in the intracellular environment (Alvarez-Sieiro et al., 2014). The strain was most effective to degrade 33-mer peptide and was resistant to simulated gastrointestinal stress. Further, pre-clinical trials of these strains are still pending that need to confirm their actions against gluten.

### Probiotics and Clinical Trials in CD Patients

Inspite of a number of *in-vitro* and *in-vivo* preclinical studies on probiotics in CD, there are scarcely available data for human trials. An exploratory trial of probiotic *Bifidobacterium infantis* natren life start strain was proved to alleviate symptoms in untreated CD but was not beneficial to strengthen intestinal permeability (Smecuol et al., 2013). The effects of this probiotic proved helpful for U-CD patients with regard to gastrointestinal symptoms and serological markers. Similarly, a recent double blind randomized-placebo controlled intervention trial of *Bifidobacterium longum* CECT 7347 improved growth related parameters in Spanish children under study thereby improving the efficacy of GFD (Olivares et al., 2014). This strain affected lymphocyte subsets which might contribute to recovery from mucosal inflammation. Administration of this probiotic modulated the intestinal microbiota with decrease in total copy number of microbes and decrease in *Bacteroids fragilis* that further correlated with decrease in secretory IgA evaluated from stool samples. These correlations presented the recovery of intestinal mucosa after administration of *B. longum.* Probiotic intervention with two strains, *Bifidobacterium breve* BR03 and *Bifidobacterium breve* B632 was given to CD patients in a very recent study (Klemenak et al., 2015). This 3 months intervention depicted a positive effect in decreasing TNFα production in CD children on GFD and the effect was reversed after 3 months of trial. Along with advancement in probiotic therapies, helminth therapy is also emerging as a fruitful outcome of research on host microbe interactions (Giacomin et al., 2015). During this trial of experimental hookworm infection in subjects, intestinal microbiota structure was preserved with increase in microbial species richness even when challenged with moderate gluten. This study revealed that gluten-induced inflammation can be regulated by hookworms (Giacomin et al., 2015).

## Other Environmental Factors

The topics discussed in this section are in a stage of infancy in literature but have serious associations with human health (Lerner et al., 2017). Future studies may further validate these concerns to clarify their real picture in disease pathogenesis.

### Probiotics and Antibiotic Resistance Genes

Gene flux in bacteria is well reported (Courvalin, 2008) and plasmids of lactic acid producing bacteria contain genes responsible for resistance to tetracycline, erythromycin, chloramphenicol, lincosamide, macrolides, streptomycin, and streptogramins (Courvalin, 2008). Administration of probiotics to the patients may lead to transfer of this resistant gene pool to other microbial communities via horizontal gene transfer. There are several hypotheses about this concept demanding a valid study to reach a conclusion. Some studies have tried to address this controversy with compromised outcomes (Duranti et al., 2017) and another study has evaluated that the transfer of a tetracycline resistance gene from probiotic *L. reuteri* to bacteria in the human gut was not observed (Egervarn et al., 2010). To better clarify the hypothesis, a well-planned study similar to that of Egervarn et al. (2010) is required so as to explore the exact impact of using probiotics on resistome of human gut microbiota.

### Microbial Transglutaminase: Cause or Consequence of CD

Microbial transglutaminase (mTG) is used in the food industry for many functions. It has considerable potential to improve the texture, viscosity, elasticity and water-binding capacity of food products and is used on a large scale for better fermentation tolerance in baked goods such as breads, pastas, pastries, and tortillas (Kieliszek and Misiewicz, 2014). Transglutaminase is hypothesized as a direct modulator of gut permeability that acts by crosslinking tight junction proteins in the epithelial cells, but its effectiveness has not been proved, as yet. Here was the sense of overestimating mTG over the gliadin (Lerner and Matthias, 2015), because gliadin itself is such a strong immunotoxic agent that can disrupt tight junction assembly of epithelial cells. mTG crosslinks numerous ingredients in the food industry, emulsifying them and potentially facilitate their passage through the tight junction (Lerner and Matthias, 2015). Thus, this property of mTG can be correlated with cross-linking of gluten peptides that can facilitate their passage through the tight junctions. The concept about mTG to cross-link, emulsify and throw the tight junction proteins out, need to be validated yet. To the best of literature search, except for the hypothesized theories and predictions, emulsifiers are not experimentally proved to affect the tight junction leakage till date, instead emulsifiers/ mTG might have allowed passage of gluten, other antigens and food ingredients from the intestinal lumen in order to produce inflammation and thus tight junction dysfunction. At present, there is an urgent need to clarify this controversy of mTG in relation to CD pathogenesis. So, if it is not evidence based, before hypothesizing such a fact, there might be a need of clarity about the amount of enzyme activity left after fermentation and other industrial processes. So the questions may arise in context with whether this enzyme remain active even after industrial go through and is also able to tolerate variable pH from mouth to small intestine to remain active in intestinal micro-environment so as to cause damage to human intestine? Further, animal studies or *in-vitro* studies may be planned to check the role of gliadins in the presence or absence of mTG and evaluating the gene expression of inflammatory and tight junction proteins in intestinal cells and such studies would be an important step forward.

An important study has recently unraveled immunological responses caused by mTG in CD patients (Matthias et al., 2016) and has shown the importance of mTG as an immunogenic agent for CD patients. For the first time, they report that mTG stimulates human immune system to produce specific antibodies. Importantly, immunogenicity of those antibodies was reported only in CD patients and not in nonceliac, symptomatic children, pediatric and adult control groups. Although, anti mTG neo-epitope antibodies levels positively correlate to the degree of the intestinal injury in CD but it cannot be interpreted that mTG is the cause of injury. Rather, it can be concluded that mTG ingested via food products get exposed to the immune cells in gliadin/ dysbiosis induced inflamed intestine of CD patients. Thus, immune response produced against mTG might leads to production of specific antibodies that are reported by Matthias et al. (2016) in CD patients but not in controls. Therefore, mTG or mTG induced immune responses as a cause or a consequence of CD, needs to be validated further.

## Functional Foods

Since, probiotic bacteria are known to have protective effects in different manners, thus these are expected to ferment the foods that can be safe for CD patients. Functional foods are the foods with health-promotion or disease prevention effects that include processed food or foods prepared with health-promoting additives (Nagpal et al., 2012). Fermented foods with live cultures are also considered functional foods with probiotic benefits (Stanton et al., 2005).

Lactic acid bacteria under specific processing conditions have the capacity to effectively hydrolyse the major gluten protein gliadin (Di Cagno et al., 2004; De Angelis et al., 2006). Selected bacteria *Lactobacillus sanfranciscensis* 7A, *Lactobacillus alimentarius* 15M, *Lactobacillus hilgardii* 51B and *Lactobacillus brevis* 14G completely hydrolyzed gliadins during fermentation of a mixture of millet and buckwheat flours, wheat and non-toxic oat (Di Cagno et al., 2004). Forty-six strains of sourdough LAB were also screened for their proteolytic activity and the sourdough cultures with *Lactobacillus sanfranciscensis* LS40, LS41, *Lactobacillus plantarum* CF1 were used for the manufacture of gluten free bread (Di Cagno et al., 2008). Studies have shown that the proteolytic action of bacteria can eliminate traces of gluten component from the contaminated gluten free food products (Gobbetti et al., 2007). Several strains worked differently to degrade 31–43 and 62–75 alpha-gliadin fragments, whereas the 57–89 peptide degradation depends upon their genetic information to produce peptidases (Gerez et al., 2008). Trials of fermented foods in CD subjects were also proved safe with recovery from intestinal inflammation (Calasso et al., 2012; Di Cagno et al., 2010). A 60-day baked diet made from hydrolyzed wheat flour with sourdough *Lactobacilli* and fungal proteases was not toxic to subjects with CD (Greco et al., 2011). The digestion of protein increased when fermentation is followed by use of intermediate content of gluten rather than whole gluten (Rizzello et al., 2014). The probiotic VSL#3 also decreased the toxicity of wheat flour when fermented for a longer duration (De Angelis et al., 2006). Gliadins predigested by VSL#3 lead decreased intestinal mucosal permeability by decreasing reorganization of the F-actin (Clemente et al., 2003; Drago et al., 2006). Inspite of extensive research on glutenases and fermented products to reduce/ elininate gluten content, there is lack of such products in market. The reason may rely in the safety of such products because even the traces of left over gluten escaping the fermentation may be harmful for patients. Such controversy was reported in the pilot study conducted by Di Cagno et al. (2010) in which eight patients were challenged with daily consumption of 200 g of wheat baked goods equivalent to 10 g of native gluten. Subsequently, out of these eight patients, 1 patient interrupted the trial after 15 days and another has done so after 30 days only due to difficulties in the compliance of the daily consumption (Di Cagno et al., 2010). This study concluded that the product was safe except for one patient who could not continue the trial. Another study also had similar findings that some of the patients could not complete a trial of baked goods for 2 months. Some patients discontinued the trial due to disease symptoms or other had developed subtotal villus atrophy (Greco et al., 2011). The reason for lack of commercialization of such products may be due to excecution of formal trials for a longer duration even for more than 2 month period. Moreover, the products do not look safe untill there is complete elimination of gluten content from such products. The quality check of such baked products should be strict enough to valiadte the comlete eliminiation of gluten content from the wheat.

Thus, relatively, the development of gluten free cereals or use of pseudocereals will be most relevant to maintain nutritional values and safety for T-CD subjects. Further, the use of enzyme therapies or functional foods may prove to be harmful because even traces of gluten peptides, if left undigested in a fermented product, may lead to reoccurrence of disease in the CD patients.

## Prospective and Conclusion

**FIGURE 2:**
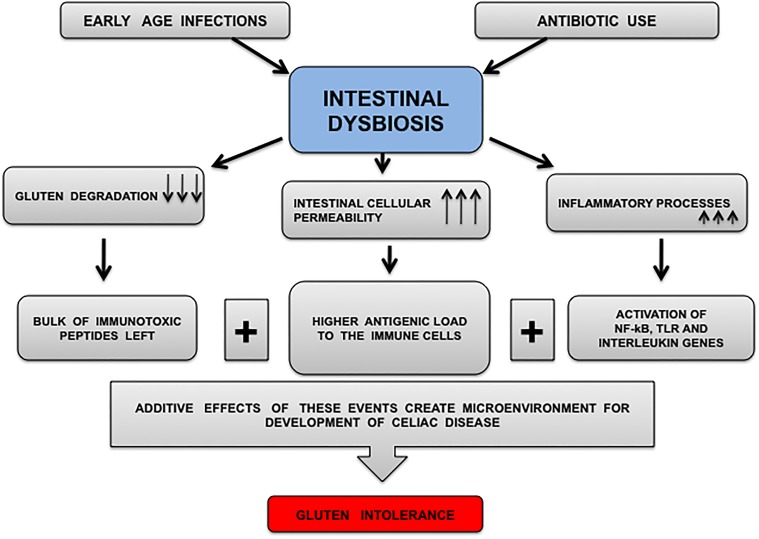
Diagrammatic representation of microenvironment proposed for CD development. Infections and antibiotic intake produce CD prone environment that affects commensals communities. Decreased number and diversity of commensals results in immune dysregulation because lesser numbers of commensals (microbes secreting glutenases) may lead to inefficient gluten digestion followed by decreased intestinal barrier function and leaving behind significant amount of intact immunotoxic peptides for immune activation. The arrows marking downward represent decrease in event whereas arrows marked upward represent increase in an event.

CD is caused by interplay between gluten, genetic factors and environmental factors (gut microbiota). Genetic factors proved to be associated with CD in a country may not define the disease risk in other country. It was reported that children possessing HLA haplotype DR3–DQ2 or DR4–DQ8 are at increased risk of CD. The relative disease risk imparted by such genes was reported more in Sweden than in United States, Finland and Germany. This highlights the importance of environmental factors in development and pathogenesis of CD (Liu et al., 2014). This fact is deriving attention toward the importance of factors other than genetic factors as a disease trigger. Such factors can be different dietary habits of different populations. As gluten is the causal antigen responsible for CD, the populations relying upon gluten diets may have greater risk of disease development. Data is scarce on this part of controversy, but depending upon existing literature, it can be hypothesized that environmental factor (GI infections, antibiotics, intestinal dysbiosis) allow gluten to trigger CD irrespective of genetic factors. In the absence of gluten, the effect of such environmental factors for development of CD is less or negligible where wheat is not a staple food. India is a diverse country and different geographical regions have different dietary habits. Wheat is the staple food for north Indian population, whereas north-east Indian population rely on rice, south Indian population rely on rice based products and sea foods. Thus, accordingly is the prevalence of CD in different populations of India. Mean daily wheat intake was highest in northern (455 g) compared with northeastern (37 g) or southern part (25 g), whereas daily rice intake showed an inverse pattern. The population prevalence of genes determining HLA-DQ2 and/or -DQ8 expression was 38.1% in northern, 31.4% in northeastern, and 36.4% in southern India. The prevalence of celiac autoantibodies was observed 1.23% in north India, 0.87% in north east India, and 0.10% in south India (*P* < 0.0001). Such diversity in dietary habits is not reported as much as in India than in other populations of the world so the phenomenon may not be observed well in other populations of world or there is lack of such well planned prevalence study based on dietary habits and risk genetic factors. In this recent study from different regions of India, gluten intake was correlated to prevalence of CD instead of HLA genetics (Ramakrishna et al., 2016). It can be concluded that there might be the additive effects of gluten and infection or antibiotic induced dysbiosis that interfere with mucosal immune homeostasis to develop this complex disorder (Figure 2). Although, a different pattern of prevalence of CD was also obsereved in different European countries i.e., 4% in Spain (Cilleruelo et al., 2015), 2.4% in Finland (Mustalahti et al., 2010), 0.9% in Germany (Laass et al., 2015), 0.6% in Northern Sweden and Hungary (Enroth et al., 2013; Burger et al., 2014), lesser in Scotland 0.01% (White et al., 2013) and least in Netherlands 0.006% (Burger et al., 2014) but in these countries, the hypothesis of environmental factors may not be validated due to lack of well planned studies emphasized on dietary habits and genetics in relation to disease prevalence.

Human leukocyte antigen and non-HLA genes are best used to identify individuals at risk of CD (Romanos et al., 2009), but genetics in CD, either defined by HLA or non-HLA genes seems secondary to the environmental factors. Therefore, the environmental factors that influence gut barrier integrity might have primary role in incidence, progression and management of CD rather than genetics (Figure 1). Apart from this, gluten is considered as environmental trigger of the disease whereas infections and use of antibiotics need to be addressed equally as important factors in pathogenesis of CD.

### Clinical Implications

Infections and antibiotic treatment in the early stages of life is the contributing factor for dysbiosis, as a trigger for CD (Cinova et al., 2011; Pozo-Rubio et al., 2013; Sanchez et al., 2013; Canova et al., 2014; Shmidt et al., 2014), that may be reversed by modulating gut microbiota. Decreased number of *Lactobacilli* and *Bifidobacteria* were reported in CD as compared to healthy individuals, where the former has potential to degrade immunotoxic peptides of gliadin while later is well known for its protective effects exerted on gliadin induced inflammation. Therefore, imbalance with these bacterial species can be supposed to influence gluten digestion and inflammatory state of intestine (Table 1).

Apart from intestinal microbiome, some reports about oral microbiome prove its crucial role in the digestion of dietary gluten (Helmerhorst et al., 2010; Zamakhchari et al., 2011; Fernandez-Feo et al., 2013). Oral microbiome might be a determining factor for composition of intestinal microbiome. In addition, very recently Francavilla et al. (2014) reported that salivary microbiota is associated with CD (Francavilla et al., 2014), so the role of oral microbiota as the gluten degraders cannot be ignored.

Celiac disease is now well studied for oral and intestinal dysbiosis. In such a condition, it is advisable that along with GFD, administrating the subjects with probiotics may help in improving intestinal barrier function by immunomodulations as well as gut microbiota modulations, improving quality of life because GFD itself cannot restore intestinal microbiome (Wacklin et al., 2014) and small-intestinal mucosa (Tuire et al., 2012). In contrast, probiotics may suppress disease complications in U-CD subjects also because LABs may act to degrade immunotoxic gluten peptides, thereby creating tolerance to gluten. Another aspect, that short chain fatty acids produced by LABs possess anti-inflammatory potential, potential of intestinal barrier function restoration and potential to modulate regulatory T cell function at intestinal mucosa (Mariadason et al., 1997; Peng et al., 2007; Furusawa et al., 2013). At present, instead of enzyme therapies and other approaches, probiotic therapy seems as the most applicable and safe biotherapy like a multipurpose sword against CD.

## Critical Appraisal of Study and Future Research Targets

Microbes, now an important part in pathology of U-CD and T-CD patients but rare efforts have been made to modulate the intestinal microbiota in a health favorable manner (Duenas et al., 2015). Diet is the modulator of gut microbiota (Ghosh et al., 2014; Ojeda et al., 2015), but except several reports on pre and post GFD examination of gut microbiota, lesser is known about the modulation in dietary therapy and its impact on intestinal microbes. Although, sequencing technologies have been frequently manipulated to understand diet-microbe interactions and its effects on host physiology (Suez et al., 2014) but in CD it needs more advancements. For example, according to a very recent report, effect of an Italian-style GFD was observed in the patients who were following African-style GFD for at least 2 years. Salivary microbiota and metabolome were observed with significant differences suggesting about metabolic dysfunction after switching to Italian-style dietary habits. This switching had lead to enrichment of *Granulicatella, Porphyromonas* and *Neisseria* while decreased counts of *Clostridium, Prevotella* and *Veillonella* (Ercolini et al., 2015). At present, as GFD is the only option for patients, therefore, efforts should be made to modulate the GFD in such a manner that it should promote the growth of beneficial microbes and should promote a better host metabolic control rather than growth of pathogens leading to metabolic deregulation. By modulating the GFD, post-GFD complications may also be best managed and GFD may remain as the best treatment option of CD ever. The kind of diet based interventions being used by Ercolini et al. (2015) are not related to the risk of horizontal gene transfer and antibiotic resistance that may be a probable outcome of using probiotics. Some of the diet interventions may shift the microbial balance in a positive way thereby encouraging the growth of beneficial microbes with simultaneous decrease in the counts of pathogens. Specific dietary approaches or prebiotics will only encourage the growth of indigenous beneficial microbes and is an approach step forward to the probiotics.

A combination of probiotics having specific properties can be recommended because as per the reported human trials, patients were administered with strains of *Bifidobacteria* only (Smecuol et al., 2013; Olivares et al., 2014; Klemenak et al., 2015), but different microbes possessing specific beneficial properties and glutenase producing microbes should be included in a panel of probiotic supplement to achieve desired goals.

As microbial dysbiosis is reported by several studies in CD (Sellitto et al., 2012), with an aim to find dysbiosis as a cause or consequence of disease, from a subset of 22 at risk individuals. It has been concluded that HLA-DQ2 genotype selects early intestinal microbiota composition in infants at high risk of developing CD (Olivares et al., 2015) but a recent Indian study on 23,331 adults supported the importance of other factors rather than genetics, in which HLA genes were not associated with prevalence of disease (Ramakrishna et al., 2016). To better understand the controversial status and contribution of interacting environmental factors, microbiota and host genetics in CD, future research in this area should focus on the impact of a change in particular type of microbiota on disease specific markers in individuals with and without genetics susceptibilities. In this pursuit, whole genome sequencing of microbes, metagenomics and transcriptomics would be helpful (Franzosa et al., 2014). In addition, prevalence and types of infections associated with CD need to be explored. The future studies should evaluate the impact of such infections on disease outcome (Shoaie et al., 2015). Impact of different types of antibiotics in creating disease susceptible environment should be focused for clarity on the role of environmental factors. Due to some prime importance, the role of specific biologically active dietary components can also be studied in ameliorating the effects of dysbiosis (Zhou et al., 2015).

## Author Contributions

AC wrote the article. HY and SJ proposed and helped in editing this article. SB and DD edited the article. All authors have read and approved the final manuscript.

## Conflict of Interest Statement

The authors declare that the research was conducted in the absence of any commercial or financial relationships that could be construed as a potential conflict of interest.

## Abbreviations

AN-PEP: *Aspergillus niger* prolyl endoprotease
Caco-2: Colorectal Adenocarcinoma
CD: Celiac Disease
GFD: Gluten Free Diet
HCs: Healthy Controls
HLA: human leukocyte antigen
HMW-GS: High molecular weight-glutenin subunits
LAB: Lactic Acid Bacteria
mTG: Microbial transglutaminase
PEP: Prolyl Endoprotease
T-CD: Treated-CD
TLR: Toll-like Receptor
U-CD: Untreated-CD
